# A review on the role of HAND2-AS1 in cancer

**DOI:** 10.1007/s10238-023-01092-3

**Published:** 2023-05-19

**Authors:** Soudeh Ghafouri-Fard, Bashdar Mahmud Hussen, Snur Rasool Abdullah, Maryam Dadyar, Mohammad Taheri, Arda Kiani

**Affiliations:** 1 Department of Medical Genetics, School of Medicine, Shahid Beheshti University of Medical Sciences, Tehran, Iran; 2 Department of Clinical Analysis, College of Pharmacy, Hawler Medical University, Erbil, Kurdistan Region Iraq; 3 Department of Medical Laboratory Science, Lebanese French University, Erbil, Kurdistan Region Iraq; 4 Phytochemistry Research Center, Shahid Beheshti University of Medical Sciences, Tehran, Iran; 5 Institute of Human Genetics, Jena University Hospital, Jena, Germany; 6 Urology and Nephrology Research Center, Shahid Beheshti University of Medical Sciences, Tehran, Iran; 7 Loghman Hakim Hospital, Shahid Beheshti University of Medical Sciences, Tehran, Iran; 8 Tehran Lung Research and Developmental Center, Shahid Beheshti University of Medical Sciences, Tehran, Iran

**Keywords:** HAND2-AS1, Cancer, lncRNA, Tumor, Metastasis, Biomarker

## Abstract

HAND2 antisense RNA 1 (HAND2-AS1) is a newly recognized lncRNA encoded by a gene on 4q34.1. This lncRNA has 10 exons and is predicted to have a positive effect on expression of certain genes. HAND2-AS1 is mainly considered as a tumor suppressive lncRNA in different tissues. Moreover, HAND2-AS1 has been shown to regulate expression of several targets with possible roles in the carcinogenesis through serving as a sponge for miRNAs. This lncRNA can also influence activity of BMP, TGF-β1, JAK/STAT and PI3K/Akt pathways. Down-regulation of HAND2-AS1 in tumor tissues has been associated with larger tumor size, higher tumor grade, higher chance of metastasis and poor clinical outcome. The present study aims at summarization of the impact of HAND2-AS1 in the carcinogenesis and its potential in cancer diagnosis or prediction of cancer prognosis.

## Introduction

Long non-coding RNAs (lncRNAs) are a group of transcripts with established roles in the fundamental process of cell physiology. Abnormal expressions of these transcripts have been linked with the development of several disorders, particularly cancers [1, 2]. This is mainly attributed to the important effects of lncRNAs in the regulation of cell cycle transition, cell proliferation, apoptosis and differentiation as well as stemness [3, 4]. Through interacting with mRNAs, DNA molecules, proteins, and miRNAs, lncRNAs influence expression of genes at almost all possible levels [5]. At epigenetic level, they mediate chromatin modification and DNA methylation. At transcriptional level, they mainly interact with transcription factors and DNA sequences. Moreover, they modulate mRNA processing; thus, they are involved in the regulation of gene expression at the post-transcriptional level. Finally, they have functional interactions with proteins to affect translational and post-translational mechanisms [5].

**Fig. 1:**
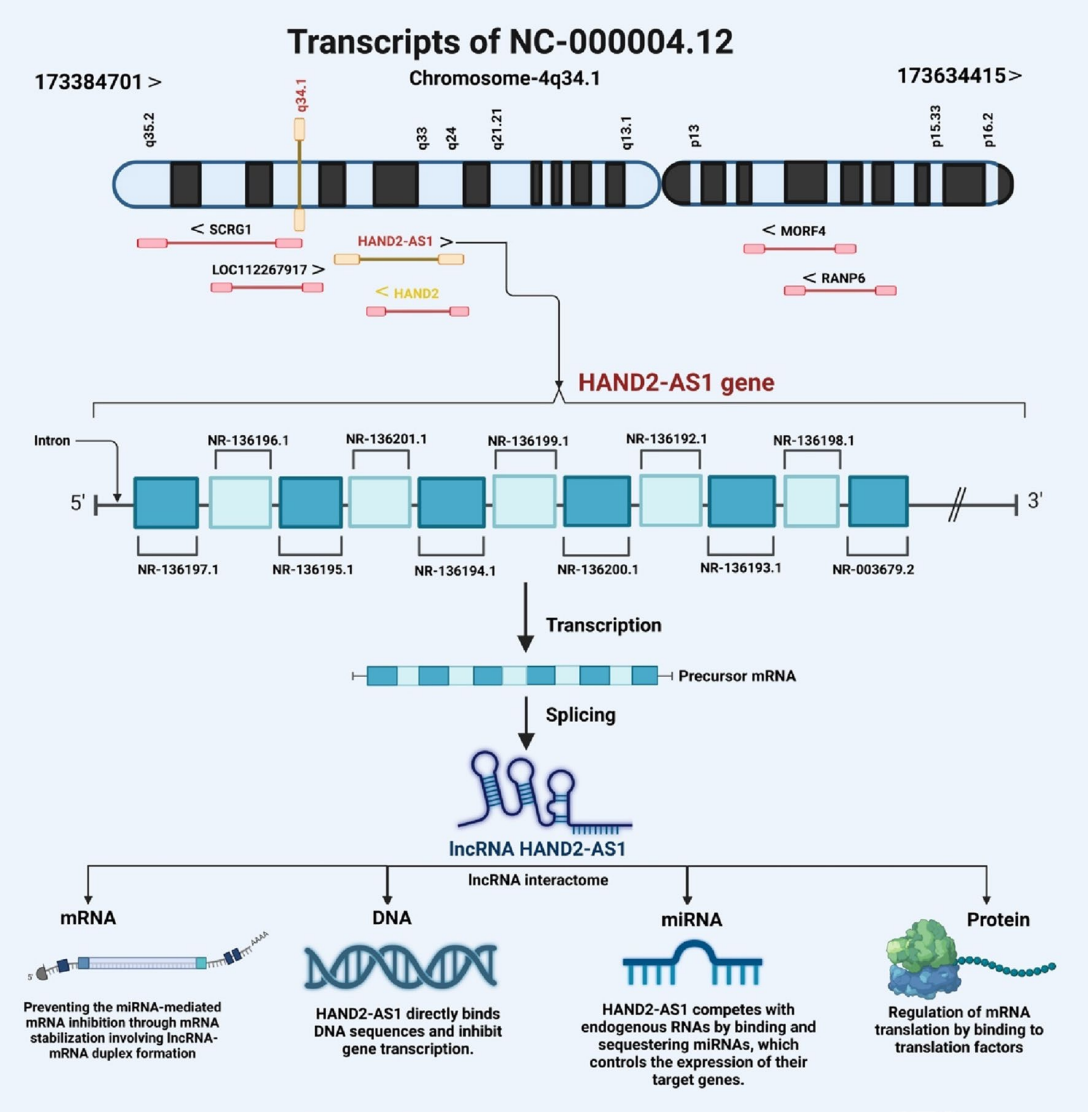
HAND2-AS1 is located on human chromosome 4. It has at least 11 transcripts (based on the NCBI database). HAND2-AS1 has various functions at different levels, such as DNA, mRNA, and protein levels. The early onset and poor prognosis of several tumor types are also related to lower HAND2-AS1 expression

HAND2 antisense RNA 1 (HAND2-AS1) is a newly recognized lncRNA encoded by a gene on 4q34.1, in an opposite direction to HAND2 gene. Transcription of HAND2AS1 might regulate expression of HAND2 (class A basic helix–loop–helix protein 26), a regulator of heart development which is involved in the pathogenesis of cardiomyopathy [6]. HAND2-AS1 has 10 exons and is predicted to have a positive effect on the expression of certain genes. Moreover, it has been mainly detected in the cytoplasm [6]. Several alternative spliced variants have been identified for this lncRNA with different sets of exons (Fig. 1).

Although the main physiological role of HAND2-AS1 is regulation of cardiac development through influencing expression of HAND2 [6], most of studies conducted on the function of this lncRNA have been performed in the context of cancer. The present study aims at summarization of the impact of HAND2-AS1 in the carcinogenesis and its potential in cancer diagnosis or prediction of cancer prognosis. We have organized the obtained evidence from literature into three distinct subtitles, including cell line studies, studies in xenograft models of cancer and studies in clinical samples.

### Search strategy

We searched PubMed and Google Scholar databases with key words "HAND2-AS1" AND "Cancer" OR "Tumor." Then, the relevance of the retrieved articles was assessed through reviewing the abstract and full text of the manuscript. After reviewing 50 articles, a total of 31 articles were selected for inclusion in the manuscript. The inclusion criteria were: 1. type of publication: original study, 2. language: English, 3. study design: in vitro and/or studies in clinical samples.

### In vitro studies

Function of HAND2-AS1 has been assessed in a variety of cancer cell lines mainly though knock-in studies. These studies have used some techniques to enhance expression of HAND2-AS1 in a variety of cancer cell lines. Then, they have assessed the functional consequences of over-expression of HAND2-AS1 in these cell lines.

### Colorectal cancer

Forced overexpression of HAND2-AS1 in colorectal cancer cells has reduced the proliferation ability and invasive properties of these cells. Mechanistically, HAND2-AS1 can serve as a molecular sponge for miR-1275. This miRNA can target KLF14; therefore, HAND2-AS1 suppresses progression of colorectal cancer via enhancing KLF14 expression [7]. This study has confirmed the tumor suppressor role of HAND2-AS1 in colorectal cancer.

HAND2-AS1 has also been found to be down-regulated in 5-fluororacil-resistant colorectal cancer cells, parallel with down-regulation of PDCD4 and up-regulation of miR-20a. HAND2-AS1 could suppress 5-fluororacil resistance, cell proliferation, migratory potential and invasive properties and promote cells apoptosis in 5-fluororacil-resistant colorectal cancer cells. Mechanistically, HAND2-AS1 acts as a sponge for miR-20a to modulate levels of PDCD4 [8]. Thus, HAND2-AS1 is a target for modulating resistance of colorectal cancer cells to 5-fluororacil.

### Esophageal cancer

Up-regulation of HAND2-AS1 in squamous cell carcinoma cells of esophagus has resulted in downregulation of miR-21 and inhibition of cell proliferation, migration, and invasive aptitude of these cells. miR-21 up-regulation could not influence expression level of HAND2-AS1, yet it could attenuate the suppressive impact of HAND2-AS1 up-regulation on these cells [9]. Cumulatively, tumor suppressive roles of HAND2-AS1 in esophageal cancer are exerted through regulation of miR-21 expression.

### Lung cancer

**Fig. 2:**
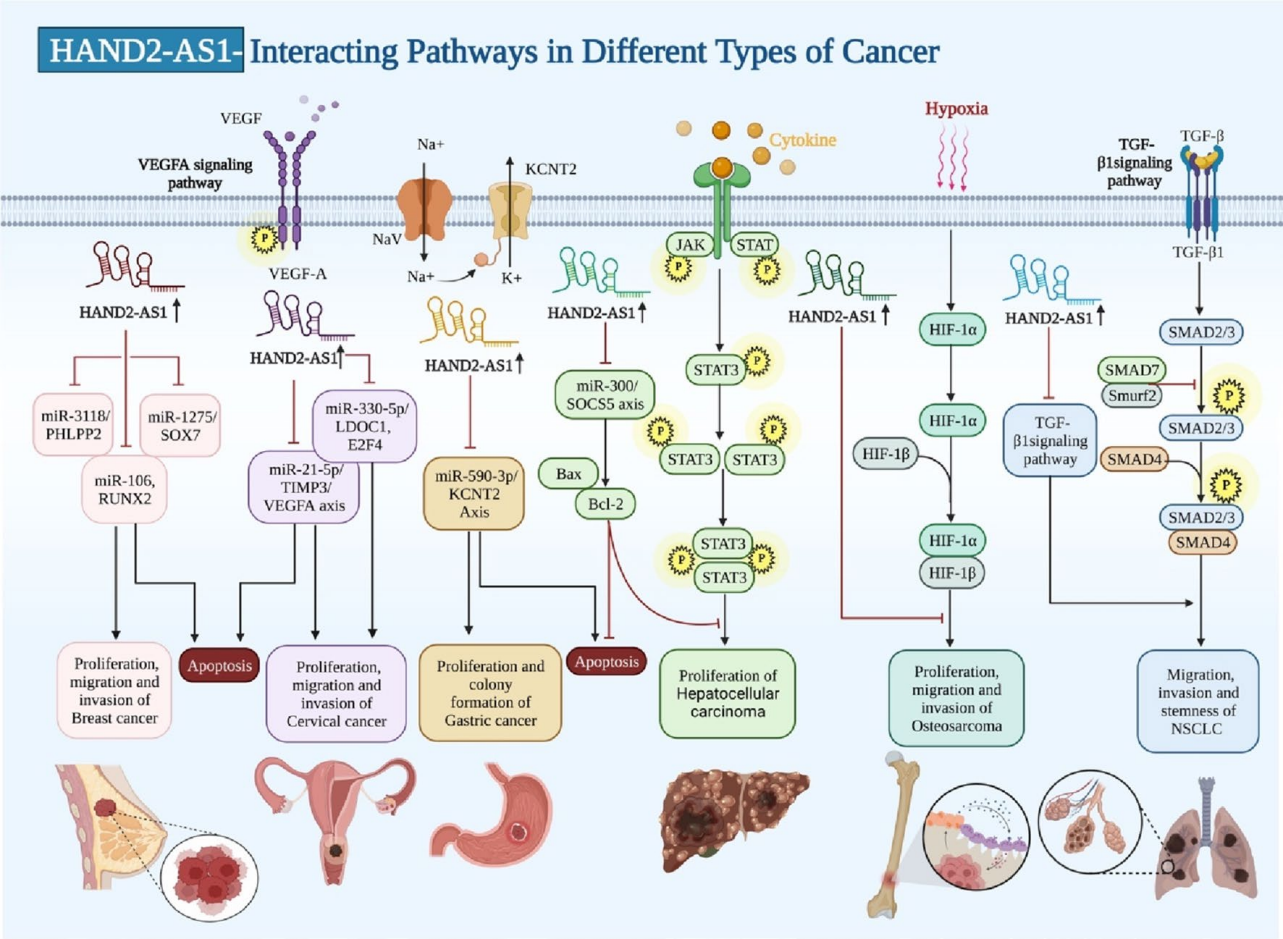
Impacts of HAND2-AS1 on different types of cancer. HAND2-AS1 performs its function as a tumor suppressor by either sponging various types of miRNAs or by suppressing the activity of various proteins that are carcinogenic

An experiment in lung cancer cells has shown that HAND2-AS1 over-expression blocks migration and invasive aptitude and stemness features of cells, while TGF-β1 has the opposite effects. Up-regulation of HAND2-AS1 in these cells has led to down-regulation of TGF-β1, while TGF-β1 has failed to affect expression of HAND2-AS1 (Fig. 2). However, TGF-β1 could attenuate the inhibitory effect of HAND2-AS1 up-regulation on invasive properties of lung cancer cells. Therefore, HAND2-AS1 has a role in the regulation of migratory potential, invasion, and stemness of lung cancer cells via interacting with TGF-β1 [10].

### Hepatocellular carcinoma

In hepatocellular carcinoma, HAND2-AS1 reduces viability of cancer cells through modulation of miR-300/SOCS5 axis [11]. Moreover, it can suppress cancer cell proliferation through down-regulation of RUNX2 [12] and ROCK2 [13]. Besides, HAND2-AS1 suppresses proliferation of liver cancer cells and their migration through enhancing expression of SOCS5 and inactivating the JAK-STAT signaling [14]. Thus, malignant feature of hepatocellular carcinoma cells is modulated by HAND2-AS1 via different mechanisms.

In spite of the observed down-regulation of HAND2-AS1 in most of cancer cells, this lncRNA has been found to be over-expressed in liver cancer stem cells (CSCs). Most notably, HAND2-AS1 has a fundamental role in the maintenance of self-renewal of these CSCs and is required for development of hepatocellular carcinoma. From a mechanistical point of view, HAND2-AS1 can recruit the INO80 chromatin-remodeling complex to the BMPR1A promoter to induce expression of this gene and subsequently activate BMP signaling [15]. Table 1 shows the role of HAND2-AS1 in different cancer cell lines.

**Table 1:** Effects of HAND2-AS1 in cancer based on cell line studies

Cancer type	Targets/regulators and signaling pathways	Assessed cell lines	Function	References
Colorectal cancer	miR-1275/KLF14	HT29, SW480, SW620, LoVo and HCT116 SW480 HCT116 cells	↑↑ HAND2-AS1: ↑ Inhibition cell proliferation rate, ↓ Invasion	[7]
Colorectal cancer	miR-20a/PDCD4 axis	NCM460/HCT116 and SW480	↑HAND2-AS1: ↓5-FU resistance, ↓cell proliferation, ↓Migration ↓Invasion, ↑Cell apoptosis	[8]
Esophagus squamous cell carcinoma	miR‐21	KYSE‐30 /KYSE70	↑↑ HAND2-AS1: ↓Proliferation, ↓Migration, ↓Invasion	[9]
Non-small cell lung cancer	TGF-β1signaling pathway	NCI-H1581 [H1581] and NCI-H1993 [H1993]	↑ HAND2-AS1: ↓ Migration, ↓Invasion, ↓ stemness	[10]
Non-small cell lung cancer	PI3K/Akt pathway	NCI-H23, NCI-H522 and BEAS-2B	↑↑ HAND2-AS1: ↓Cell proliferation, ↑ Apoptosis	[16]
Non-small cell lung cancer	WTAPP1	H1581 and H1993	↑↑ HAND2-AS1: ↓ Invasion, ↓Migration	[17]
Hepatocellular carcinoma	miR-300/SOCS5 axis Bax/Bcl-2	HEK-293 T/HL-7702/Huh-6, Huh-7 and SK-HEP-1	↑↑ HAND2-AS1: ↓ Proliferation	[11]
Hepatocellular carcinoma	RUNX2	SNU-398/SNU-182	↑HAND2-AS1: ↓ Cell viability, ↓Migration, ↓Invasion, ↓Proliferation	[12]
Hepatocellular carcinoma	ROCK2	SNU‑398/THLE‑ 3	↑↑ HAND2-AS1: ↓ Cell migration, ↓Proliferation, ↓Invasion	[13]
Liver cancer stem cells	INO80 complex/BMPR1A/BMP signaling	Hep3B, Huh7, and PLC/PRF/5	HAND2-AS1: maintenance of self-renewal of CSCs	[15]
Chronic myeloid leukemia	miR-1275	KCL22 / K562	↑ HAND2-AS1: ↓ Cell Proliferation, ↓ Invasion, ↑Cell Apoptosis	[18]
Osteosarcoma	HIF1α	MG-63, SAOS-2, U-2OS, HOS, SW1353	↑↑ HAND2-AS1: ↓Proliferation, ↓Migration, ↓Invasion	[19]
Osteosarcoma	GLUT1	MG‑63 and SAOS‑2/hFOB	↑ HAND2-AS1: ↓ Proliferation	[20]
Gastric Cancer	miR-590-3p/KCNT2 Axis	GES-1/SGC-7901 and BGC-823	↑↑ HAND2-AS1: ↓ Proliferation, ↓Colony formation, ↑ Apoptosis	[21]
Gastric cancer	miR-184 /HIF3A	NCI-N87/ AGS	↑ HAND2-AS1: ↓Cell proliferation, ↓Migration, ↓Invasion	[22]
Gastric cancer	miR-769–5p/TCEAL7 axis	HGC-27 /MGC-803	↑↑ HAND2-AS1: ↓Cell proliferation, ↓Invasion, cell cycle arrest at G0/G1 phase ΔHADN2-AS1: ↑↑Cell proliferation, ↑↑Invasion	[23]
Endometrioid endometrial carcinoma	NMU (neuromedinU)	HEC1-A, HEC1-B, AN3CA, KLE, RL95-2	↑ HAND2-AS1: ↓ Migration, ↓Invasion ↑↑Hand2-AS1: ↓ Migration, ↓Invasion	[24]
Ovarian cancer	BCL2L11/ miR‐340‐5p	ES‐2, Caov‐3, SKOV3, OVCAR‐3	↑↑ HAND2-AS1: ↓Invasion, ↓Migration, ↓Cell proliferation, ↑Cell apoptosis	[25]
Ovarian cancer DDP‑resistant	miR‑106a/PTEN axis	SKOV3 SKOV3/DDP	↑HAND2-AS1: ↓ Resistance to DDP	[26]
Bladder cancer	RARB/ miR‑146/ Caspase 3	5637 (HTB-9™), RT4 (HTB-4™) and J82 (HTB-1™)	↑ HAND2-AS1: ↓ Invasion, ↑ Apoptosis, ↑ Regression	[27]
Cervical squamous cell carcinoma	ROCK1	C‑33 A (HPV negative) SiHa (HPV positive) HCvEpC(HPV negative) Ect1/E6E7(HPV positive)	↑↑ HAND2-AS1: ↓ cell proliferation, ↓Migration, ↓Invasion	[28]
Cervical cancer	E2F4	H8 and 4	↑↑ HAND2-AS1: ↓proliferation, ↓Colony formation, ↓Migration, ↓Invasion	[29]
Cervical cancer	miR-21-5p/TIMP3/VEGFA axis	SiHa, CaSki, and C33A/H8	↑↑ HAND2-AS1: ↓cell proliferation, ↓Migration, ↓Invasion, ↑ Cell apoptosis	[30]
Cervical cancer	miR‑330‑5p/LDOC1	(HeLa),Ca Ski,C-33A H1HeLa,(HUCEC)	↑↑ HAND2-AS1: ↓Proliferation, ↓Migration ↓Invasion, ↓tumor formation, ↓Metastasis	[31]
Breast Cancer	miR-106a-5p	TNBC/MCF10A	↑HAND2-AS1: ↓ Cell viability, ↓Migration ↓Invasion, ↓Proliferation	[32]
Breast Cancer	miR-3118/PHLPP2	MDA-MB-231, MCF-7, SK-BR-3, and MDA-MB-45 MCF10A	↑↑ HAND2-AS1: ↓Proliferation, ↓Migration, ↓Invasion, ↑Apoptosis	[33]
Breast Cancer	miR-1275/SOX7	MCF10A, MDA-MB-231, MDA-MB-468, MCF-7,UACC812	↑↑ HAND2-AS1: ↓Cell viability, ↓Migration, ↓Invasion	[34]
Triple‑negative breast cancer	RUNX2	MDA‑MB‑231 and BT‑20	↑↑ HAND2-AS1: ↓ cell proliferation	[35]
Melanoma	ROCK1	C32 and SK‑MEL‑28	↑↑ HAND2-AS1: ↓cell proliferation	[36]
Glioma	CDK6	U87MG, U118MG, U251 and A172 HM	↑↑ HAND2-AS1: ↓ proliferation, ↓Invasion, ↓Migration	[37]

### Animal studies

Up-regulation of HAND2-AS1 has been shown to inhibit propagation of colorectal cancer [7] and its in vivo growth [8]. Similar effects have been reported for HAND2-As1 up-regulation in cervical, breast, ovarian, bladder, and gastric cancers (Table 2).

**Table 2:** Effects of HAND2-AS1 on growth and metastasis of cancer xenografts

Cancer type	Animal models	Function	References
Liver cancer	Orthotopic transplantation of PDX liver cells or Huh7‐Luc cells into livers of NOD‐Prkdc scid Il2rg tm1/Bcgen mice	↓ HAND2-AS1: inhibition of liver carcinogenesis	[15]
Colorectal Cancer	BALB/c nu/nu male mice subcutaneous injection with 4 × 10^6^ HCT116	↑↑ HAND2-AS1: ↓ tumor growth	[7]
Colorectal cancer	Xenograft model of colorectal cancer (nude mice)	↑↑ HAND2-AS1: ↓tumor growth, ↑apoptosis	[8]
Cervical Cancer	Xenograft model of cervical cancer (nude mice)	↑↑ HAND2-AS1: ↓tumor growth	[30]
Cervical Cancer	BALA/C nude female mice Subcutaneous injection of oe-NC + NC mimic, oe-HAND2-AS1 + NC and oe-HAND2-AS1 + miR-330-5p mimic	↑↑ HAND2-AS1: ↓tumor growth ↓metastasis	[31]
Cervical Cancer	male nude mice (BALB/c) injected with the cells transfected with HAND2‐AS1 alone or in the presence of sh‐NC, sh‐E2F4, oe‐NC or oe‐C16orf74	↑↑ HAND2-AS1: ↓tumor growth	[29]
Triple negative breast cancer	nude mice injected with TNBC cells incubated with exosomes from MSCs transfected with miR-106a-5p/ subsequent injection of lentivirus containing HAND2-AS1into tumors	↑↑ HAND2-AS1: ↓tumor growth	[32]
Breast Cancer	male nude mice injected with MCF-7 cells transfected with pcDNA3.1/HAND2-AS1 and pcDNA3.1 vector	↑↑ HAND2-AS1: ↓tumor growth	[33]
Ovarian cancer	nude mice/xenograft model of ovarian cancer	↑↑ HAND2-AS1: ↓tumor growth, ↑apoptosis	[25]
Bladder cancer	Female BABL/c athymic nude mice injected with HAND2-AS1 or miR-146 expressing cells	↑↑ HAND2-AS1: ↓tumor growth	[27]
Gastric cancer	BALB/C nude mice injected with HAND2-AS1 overexpressing cells	↑↑ HAND2-AS1: ↓tumor growth	[23]

However, experiments in the humanized models of hepatocellular carcinoma have shown that antisense oligonucleotide-mediated suppression of HAND2-AS1 exerts anti-tumor activity in a synergic manner with siRNA-mediated inhibition of BMPR1A. Besides, silencing of lncHand2 or Bmpr1a in mice hepatocytes has been shown to impair BMP signaling and suppress development of liver cancer [15]. Therefore, there is a discrepancy between the bulk of evidence from most types of cancers versus hepatocellular carcinoma.

## Experiments in clinical samples

HAND2-AS1 has been demonstrated to be under-expressed in colorectal cancer tissues. Moreover, expression levels of HAND2-AS1 have been negatively correlated with metastasis and advanced stages. Besides, HAND2-AS1 down-regulation has been identified as a marker of poor prognosis [7]. Another study in colorectal cancer tissues has demonstrated down-regulation of this lncRNA in 5-fluorurcil-resistant tissues [8].

In addition, HAND2-AS1 has been reported to be downregulated in esophagus squamous cell carcinoma tissues, while miR-21 has been upregulated in these tissues compared with paired adjacent healthy tissues. Transcript levels of HAND2-AS1 and miR-21 have been inversely correlated in tumor samples but not in non-tumoral samples of these patients. Notably, authors have reported down-regulation of plasma levels of HAND2-AS1 in esophagus squamous cell carcinoma patients compared with normal persons. Besides, downregulation of plasma levels of HAND2-AS1 could distinguish early stage of this cancer from healthy status [9]. Thus, HAND2-AS1 can be regarded as a marker for early detection of cancer.

HAND2-AS1 expression has also been lower in lung cancer tissues compared with adjacent healthy tissues. Moreover, plasma levels of HAND2-AS1 have been lower in patients with lung cancer compared with controls, while TGF-β levels have been higher in these patients. Notably, plasma levels of HAND2-AS1 and TGF-β1 have been negatively correlated in patients but not in control subjects [10]. This study further supports the potential of HAND2-AS1 in cancer diagnosis.

In chronic myeloid leukemia (CML), down-regulation of HAND2-AS1 has been much prominent in accelerated and blast phases compared with chronic phase [18], indicating a putative prognostic role for this transcript. Totally, down-regulation of HAND2-AS1 has been associated with metastasis in colorectal cancer [7] and hepatocellular carcinoma [11]. Moreover, its down-regulation has been associated with advanced stage in colorectal cancer [7] and with high pathological grade in hepatocellular carcinoma [11], endometrial cancer [24], bladder cancer [27], and glioma [37]. Finally, levels of this lncRNA are associated with tumor size in non-small cell lung cancer [16], osteosarcoma [20], and melanoma [36]. Table 3 shows the role of HAND2-AS1 as described by studies in clinical samples.

**Table 3:** Role of HAND2-AS1 in cancer based on experiments in clinical samples (OS: overall survival, PTN: paired tumor and normal samples)

Cancer type	Clinical samples	Expression change in tumor tissues compared to normal tissues	Kaplan–Meier Analysis (down-regulation of HAND2-AS1)	Association of dysregulation of HAND2-AS1 with clinical data	References
Colorectal Cancer	74 PTN	Down	Shorter OS	Metastasis and advanced stage	[7]
Colorectal Cancer	27 PTN	Down	Shorter OS	5-fluorurcil resistance	[8]
Esophagus squamous cell carcinoma	66 PTN	Down	Shorter OS	–	[9]
Non-small cell lung cancer	72 PTN, 54 healthy controls	Down	Shorter OS	–	[10]
NSCLC	68 PTN	Down	Shorter OS	–	[17]
NSCLC	94 PTN	Down	Shorter OS	Tumor size	[16]
Hepatocellular carcinoma	50 PTN	Down	Shorter OS	Tumor grade, metastasis and recurrence	[11]
Hepatocellular carcinoma	78 patients 48 healthy controls	Down	–	Down regulated in early-stage	[12]
Hepatocellular carcinoma	44 HCC 38 hepatitis B	Down	–	–	[38]
Chronic myeloid leukemia	30 CML patients/ 10 healthy donors	Down	Shorter OS	Much lower in accelerated and blast phases compared with chronic phase	[18]
Triple negative breast cancer	20 PTN	Down	Shorter OS	–	[32]
Triple-negative breast cancer	63 female patients 43 healthy females	Down	–	–	[35]
Osteosarcoma	–	Down	Shorter OS	–	[19]
Osteosarcoma	48 PTN	Down	–	Tumor size, but not tumor metastasis	[20]
Gastric cancer	–	Down	–	–	[23]
Gastric Cancer	–	Down	Shorter OS	–	[21]
Gastric Adenocarcinoma	90 PTN	Down	Shorter OS	–	[22]
Endometrioid endometrial carcinoma	59 PTN	Down	Shorter OS	Tumor grade, lymph node metastasis and recurrence	[24]
Ovarian cancer	40 PTN	Down	–	–	[25]
Ovarian cancer	12 PTN	Down	–	–	[26]
Bladder cancer	32 PTN	Down	Shorter OS	Invasion and grade	[27]
Cervical squamous cell carcinoma	122 PTN	Down	–	–	[28]
Cervical cancer	58 PTN	Down	Shorter OS	–	[30]
Cervical cancer	68 PTN	Down	–	Tumor formation and lymph node metastasis	[31]
Cervical cancer	57PTN	Down	Shorter OS	–	[29]
Melanoma	56 PTN	Down	–	Tumor thickness	[36]
Glioma	56 PTN	Down	–	High pathological grade	[37]

## Discussion

This review provides a comprehensive summary of the expression level, function, and prognostic value of HAND2-AS1 in different cancers. Based on the above-mentioned data, HAND2-AS1 is mainly regarded as a tumor suppressive lncRNA in different tissues. The most possible explanation for its down-regulation in cancer tissues is hypermethylation of its promoter as demonstrated in ovarian cancer tissues [39]. Moreover, HAND2-AS1 has been shown to regulate expression of several targets with possible roles in the carcinogenesis through serving as a sponge for miRNAs. miR-1275, miR-20a, miR‐21, miR-300, miR-590-3p, miR-184, miR-769–5p, miR‐340‐5p, miR‑106a, miR‑146, miR‑330‑5p, miR-106a-5p, and miR-3118 are the main miRNAs that are influenced by this lncRNA. The interaction between HAND2-AS1 and miR-1275 has been verified in different contexts, including colorectal cancer, CML, and breast cancer. Most notably, the rs2276941 polymorphism within HAND2-AS1 has been shown to affect its binding with hsa-miR-1275 and influence the susceptibility to colorectal cancer [40].

BMP, TGF-β1, JAK/STAT, and PI3K/Akt pathways are the main cancer-related pathways being influenced by HAND2-AS1. In addition to regulation of cell proliferation and apoptosis, it can affect cancer metabolism and angiogenesis through modulation of a number of targets including GLUT1, VEGFA, and HIF1α. Most importantly, HAND2-AS1 can modulate response of cancer cells to 5-fluouracil and cisplatin.

The impact of HAND2-AS1 on the maintenance of CSCs should be further investigated, since data regarding this function are contradictory [10, 15]. Based on the importance of this population of cells in tumor metastasis and recurrence, this issue has practical significance.

Experiments in clinical samples have shown association between dysregulation of HAND2-AS1 and a number of tumor characteristics such as level of differentiation, tumor size, lymph node metastasis, and most importantly overall survival of patients. Thus, evidence regarding the prognostic role of HAND2-AS1 is ample. On the other hand, diagnostic value of this lncRNA has not been investigated thoroughly.

Although the clinical application of HAND2-AS1 as a therapeutic target has not been assessed, based on the observed down-regulation of this lncRNA in a vast variety of tumors with different origins, up-regulation of HAND2-AS1 can be regarded as a therapeutic target for several types of cancer. This can be achieved by specific epigenetic regulators that affect epigenetic marks in the promoter of this lncRNA. This therapeutic modality can also affect response of patients to conventional chemotherapeutic agents.

Finally, the impact of polymorphisms within *HAND2-AS1* gene on risk of cancer has not been investigated. These polymorphisms can affect binding of this lncRNA to certain miRNAs, thus influencing the functional roles of HAND2-AS1.

## Future perspectives

Forced up-regulation of HAND2-AS1 in cancer cells is a possible route for reduction in malignant features. Thus, the applicability and safety of this method should be assessed in clinical settings to find a novel treatment option for cancer.

## Data Availability

The analyzed data sets generated during the study are available from the corresponding author on reasonable request.
